# Hepatitis E: Genotypes, strategies to prevent and manage, and the existing knowledge gaps

**DOI:** 10.1002/jgh3.12646

**Published:** 2021-08-30

**Authors:** Lubna Kamani, Zahra Ali Padhani, Jai K Das

**Affiliations:** ^1^ Associate Professor & Director, GI Residency Program, Department of Gastroenterology Liaquat National Hospital and Medical College Karachi Pakistan; ^2^ Consultant Aga Khan University Hospital Karachi Pakistan; ^3^ Health Policy and Management, Manager (Research) Aga Khan University Hospital Karachi Pakistan; ^4^ Assistant Professor and Head, Section of Public Health and Epidemiology Aga Khan University Hospital Karachi Pakistan

**Keywords:** acute hepatitis, hepatitis E virus, hepatitis E virus treatment, hepatitis E virus vaccine, viral Hepatitis

## Abstract

Hepatitis E virus (HEV) is considered an emergent source of viral hepatitis worldwide, with an increasing burden of jaundice, liver failure, extrahepatic illnesses, and deaths in developed countries. With the scarcity of data from efficient animal models, there are still open‐ended questions about designing new models to study pathogenesis, types, virology, and evolution of these viruses. With an emphasis on available data and updates, there is still enough information to understand the HEV life cycle, pathogen interaction with the host, and the valuation of the role of vaccine and new anti‐HEV therapies. However, the World Health Organization (WHO) and the European Association for the Study of the Liver (EASL) preferred to stress prevention and control measures of HEV infections in animals, zoonotic transmission, and foodborne transmission. It is being reviewed that with current knowledge on HEV and existing prevention tools, there is an excellent room for in‐depth information about the virus strains, their replication, pathogenicity, and virulence. The current knowledge set also has gaps regarding standardized and validated diagnostic tools, efficacy and safety of the vaccine, and extrahepatic manifestations specifically in pregnant females, immunocompromised patients, and others. This review highlights the areas for more research exploration, focusing on enlisted research questions based on HEV infection to endorse the need for significant improvement in the current set of knowledge for this public health problem.

## Graphical abstract

Hepatitis E is endemic in regions, particularly South East Asia. It is the leading cause of Fulminant hepatic failure in young women with pregnancy. In the absence of definite treatment and widely available vaccine, prevention remains the key. This article mainly summarises preventive strategies and current knowledge gaps of hepatitis E.

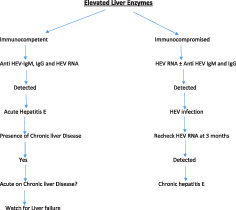

## Introduction

Hepatitis E is a liver disease caused by hepatitis E virus (HEV).^1^ It is the fifth known form of hepatitis among humans and is the one of the common causes of jaundice and acute hepatitis^2^ that leads to significant human morbidity and mortality. The disease was initially discovered in 1980s by a Russian scientist as “epidemic, non‐A, non‐B hepatitis,” an infectious, waterborne illness like hepatitis A that was common in the developing countries.^3^ Three years later, the virus was visualized in stool samples using an immune electron microscope and subsequently a viral genome was cloned and named as HEV.^4^ The virus sheds in stool of infected persons and enters the human body through intestine. It is a self‐limiting virus and resolves in 2–6 weeks, but in some cases fulminant hepatitis (acute liver failure) develops and can cause death.^5^ This review article on HEV will highlight genotypes, strategies to prevent and manage, and the existing knowledge gaps.

## Epidemiology and current burden of hepatitis E

HEV infection is an evolving pathogen in industrial countries with high prevalence in developing countries. World Health Organization (WHO) referred HEV as one of the usual utmost infections to cause acute hepatitis with an extensive spread worldwide.^6^, ^7^, ^8^ Almost 2.3 billion people get infected with HEV, which attributes 70 000 deaths annually.^6^, ^7^, ^8^, ^9^

Two different epidemiological forms have been witnessed in diverse geographical areas worldwide. These arrays are assumed to be interrelated with the spread of HEV genotypes, modes of transmission, basics of viral septicity, prevalence of disease, as well as medical indications of the infection. According to WHO and Center for Diseases Control (CDC), the clinical characteristics and epidemiology of HEV infection are principally resolute by the prime genotype, in particular, hosts and the area as illustrated by two expressions of hepatitis E infection that are associated with geographic dispersal (Table 1).^1^, ^10^, ^11^ In developing countries, the epidemiology of HEV infection indicates low seroprevalence in people under 15 years of age; however, it rises rapidly between the ages of 15 and 30 years. The incidence is variable among developing countries with reported outbreaks affecting thousands of people, specifically during flooding or monsoons with a mortality rate of 0.5–3%.^12^ HEV in developed countries shows increase in seroprevalence with age, with a peak between 30 and 50 years and is usually sporadic and affects small groups with specific food background with no transmission from human to human.^13^ In European countries, HEV is mostly acquired through local zoonotic. Animals carrying HEV virus, affecting human health includes pigs, boar, and dear.^2^ Recent data report presence of HEV 3 in dolphins in Cuba,^14^ and HEV‐4 in cattle in China, its presence was also documented in its milk even after pasteurization,^15^ which can be a risk factor for human transmission of this virus from domestic animals .

**Table 1:** Two distinct epidemiological patterns of hepatitis E infection

Characteristics	Low endemic	Highly endemic
Definition	Confirmed HEV infection in <25% of sporadic hepatitis	Waterborne outbreaks or HEV infection >25% of hepatitis
Transmission	Through animal sources and blood transfusion	Mainly through contaminated water sources and blood transfusion
Areas	Safe and clean drinking water supply	Developing countries with frequent water contamination
Genotypes	3 and 4	1 and 2
Mortality/morbidity	Low mortality and morbidity	High/intermediate mortality and morbidity
Cases	Asymptomatic and symptomatic acute hepatitis. Chronic hepatitis in immunocompromised individuals	Frequent outbreaks, asymptomatic and symptomatic acute hepatitis, acute liver failure
Effected countries	North and South America, Europe, Australia, South Africa, East Asia	Africa, Central America, South, and Central Asia

Adapted from Melgaço *et al*.^10^

In recent years, “hotspots” of HEV infection have been identified in Europe. This includes western/central Poland (seroprevalence 50%),^16^ Abruzzo, central Italy (seroprevalence 49%),^17^ Czech Republic (400 laboratory‐confirmed cases 2015),^18^ western Germany (1:616 blood donors viraemic, 2015),^17^ Scotland (1:2481 donors viraemic, 201 642),^19^ the Netherlands (1:600 blood donors viraemic, 2014),^20^ and southwest France (incidence 3–4%).^15^ There are also other areas with high levels of circulating virus, but are yet unidentified.

### Virology of hepatitis E

HEV is a small, unenveloped virus of 27–34 nm diameter with icosahedral symmetry. According to the taxonomic classification scheme, all the HEV strains are grouped under the *Hepeviridae* Family, under the genus *Orthohepevirus* (A–D, 4 species) and Piscihepevirus (A, 1 species).^10^, ^21^, ^22^ It is a highly variable virus, of which *Orthohepevirus* A species take in practically HEV variants of all mammalians with seven genotypes, out of which at least five are of human public health interest. The different HEV genotypes have a distinct geographical distribution. HEV‐1 and HEV‐2 genotypes are recognized to infect humans by causing huge waterborne outbreaks and sporadic cases in prevalent areas and these genotypes are prevalent in developing countries.^22^, ^23^ While genotypes HEV 3 and HEV 4 are distributed worldwide and have the potential to infect both animals and humans.^24^ While one report has documented HEV infection in humans with genotype HEV‐7 and HEV 8,^25^, ^26^ with genotype HEV‐7, known to chronically infect a liver transplant patient. Furthermore, cynomolgus macaques were infected using HEV genotype 8 positive samples, vulnerable to both acute and chronic infection.^13^ The genotypes 7 and 8 are deliberated to be evolving human pathogens and there is a need for more studies to screen for genotype 8 in humans.^26^

**Figure 1:**
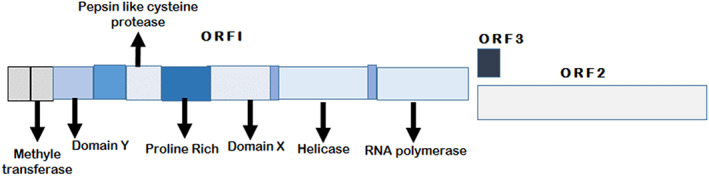
Genomic organization of HEV showing e positive sense HEV genomic RNA and a subgenomic RNA produced during replication of three ORFs, which encode ORF1, ORF2, and ORF3 proteins. The scale below shows nucleotides in thousands (Adapted from Hoofnagle *et al*.,^5^ Chandra *et al*.,^29^ Wedemeyer *et al*.^30^ and Yamashita *et al*.^31^).

There is one serotype of HEV spreading globally^27^, ^28^ that holds a 7.2 kb + ve sense RNA genome with single‐strand and three overlapping open‐reading frames (ORFs), i.e. ORF1, ORF2, ORF3, and ORF4 having distinctive roles. ORF1 encrypts an un‐structural polyprotein that possesses 1690 amino acids and entails proteins vital for RNA replication, e.g. methyltransferase, RNA helicase, RNA polymerase, papain‐like cysteine protease, and RNA‐dependent (Fig. 1). Although ORF2 is one of the most vital structural proteins for pursuing HEV vaccine development, as it encrypts capsid (protein) with neutralizing epitopes persuading antibody making in the host and reservoirs. On the other hand, ORF3 possess 123 amino acid and overlays with ORF2, and encrypts a minor multifunctional protein intricate in viral particle secretion.^22^, ^32^, ^33^ All the HEV units are susceptible to frying or boiling and become deactivated at temperatures beyond 90°C just after 5 min. To entirely deactivate HEV in the foodstuff, an internal temperature of 71°C for 20 min is mandatory^34^, ^35^ and it is reported to be vulnerable from chlorine cleansing in water supplies and fomites.^22^, ^35^ While ORF4 is novel composed of 158 amino acids in ORF1 has newly defined for HEV‐1. HEV replication is assumed to be a part of HEV replication with many viral proteins like RdRp or helicase and host factors such as eukaryotic elongation factor 1 isoform‐1 (eEF1α1) and β‐tubulin. Although the existing functional role of ORF4 in other HEV genotypes must be studied further.^36^, ^37^

### Transmission of Hepatitis E

Waterborne transmission is most common in high endemic areas with poor sanitation practices, while the zoonotic or foodborne transmission is usually caused by the consumption of raw or undercook meat from animals infected by HEV. Industrial areas account for maximum HEV transmissions, subsequently posing a significant public health problem.^30^, ^38^, ^39^ The mode of HEV transmission remains contentious, and viral infection causes are often not well known, predominantly in intermittent cases of acute hepatitis E. As in such cases, the infection may also be associated with the level of populace immunity, hygienic settings, living environments, and others dynamics.^40^ However, recent investigations support the presence of three further means of HEV transmission that includes parenteral, human to human, and perinatal transmission although these means of transmission are less frequently observed but with increasing evidence, it emphasizes prophylactic measures for the prevention of HEV infection.^38^, ^39^, ^40^, ^41^

In various animal studies, it has been identified that viral replication takes approximately 7 days after inoculation in hepatocytes and upsets up to 90% of hepatic cells causing clinical damage and illness with a typical incubation period of 21 days. As viremia occurs few days earlier to the commencement of symptoms, followed by changes in serum transaminase levels and fecal HEV excretion for a few weeks thereafter.^42^, ^43^ Regarding its pathophysiology, hepatocytes are considered as the primary site for HEV replication, but there is little evidence that reported on extrahepatic HEV replication.^44^, ^45^, ^46^ The information regarding particular receptors on hepatocytes for the binding of HEV remains imprecise. However, many studies on in vitro liver cell culture system of Huh‐7 showed that heparin sulfate proteoglycans on the exterior of the cell, principally syndecans most abundantly syndecan 1, have the potential to bind with recombinant HEV ORF2 protein expressed as VLPs. Mostly identified as a hepatotoxic virus, to some extent, HEV has been found to reproduce in other tissues causing extrahepatic indications including neurological, renal, and hematological problems^47^, ^48^, ^49^, ^50^ as it enters the body by contaminated water or food via the oral route. It is assumed that the virus makes copies inside the small intestine's epithelial cells and then reaches the liver, which is the ultimate target organ. Once in the hepatocytes, the virus proliferates and initiates liver damage.^51^

## Hepatitis E and extrahepatic manifestations

During the last decade, HEV‐associated neurological conditions that have been reported in developing countries include Bell's palsy, Guillain‐Barre syndrome, neuralgic amyotrophy, acute menginoencephalitis, brachial neuritis, ataxia, encephalitis, proximal myopathy, and acute transverse myelitis.^47^, ^49^, ^52^ HEV RNA has been detected in serum and CSF not only in acute illness but also CNS infection was found among immunosuppressed patients after organ transplantation.^53^ A study also demonstrated that cell lines of human neuronal‐derived can support HEV RNA duplication that is why it is suggested that clinicians should consider the likelihood of HEV infection in patients with neurological disorders and concurrent liver enzyme alteration.^54^ Other extrahepatic complications associated with HEV includes renal involvement like cryoglobulinemia and membrano‐proliferative glomerulonephritis. Renal impairment has been reported in solid organ transplant recipients during acute HEV infection.^2^ Hematologic manifestations include autoimmune hemolytic anemia, aplastic anemia, acute liver failure associated with pure red‐cell aplasia, severe thrombocytopenia, and cutaneous T cell lympho‐proliferative syndrome.^48^, ^55^, ^56^, ^57^ Other complications reported are acute pancreatitis, myocarditis,^58^ Henoch‐Schönlein purpura,^59^ myasthenia gravis,^60^ thyroiditis, and Grave's thyrotoxicosis.^47^ However, there is need to explore the pathological relation, mechanisms, and effective treatment because a causal relationship between these associations has not been established.

## Hepatitis E virus diagnostics

The European Association for the Study of the Liver (EASL) has suggested different methods for the diagnosis of HEV, which are as follows.^61^

### Laboratory diagnosis

The incubation period of HEV is approximately 15–60 days. HEV RNA is detected in blood and stool around 3 weeks post‐infection (with viraemia lasting approximately 3–6 weeks and shedding of virus in stool for approximately 4–6 weeks). The first appearance occurs before the onset of symptoms and at the time of clinical onset, biochemical markers elevate with first appearance of IgM antibodies (short lived), followed by IgG antibodies (long lasting).

### Molecular analysis

**Figure 2:**
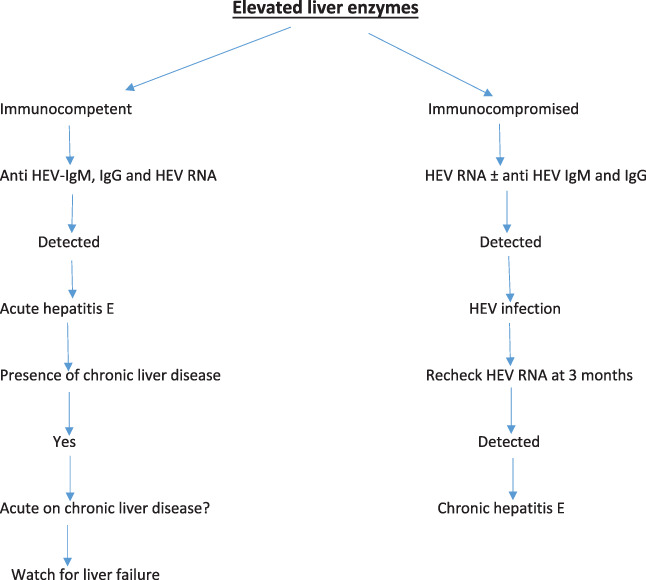
Diagnostic algorithm for HEV infection (Adapted from European Association for the Study of the Liver.^61^)

Detection of HEV RNA in stool or blood is indicative of HEV infection. In patients with suppressed immune system and chronic HEV, anti‐HEV antibodies are often undetectable, and in such cases, NATs are the only reliable means of diagnosis (Fig. 2). Many NAT‐based assays are used for the detection of HEV RNA in stool, serum, and plasma samples. It includes conventional reverse transcription PCR (RT‐PCR) and nested protocols, real‐time RT‐PCR, and transcription‐mediated amplification methods (e.g. reverse transcription loop‐mediated isothermal amplification).^62^, ^63^, ^64^ The frequently used assay methods work by targeting highly conserved regions of the genome, in particular, the region of ORF2 that overlaps ORF3, and are able to detect all four major genotypes of HEV that infect humans.^65^

### Antibody assays

Acute HEV infection can also be diagnosed by HEV NAT in combination with enzyme immunoassays by detection of anti‐HEV antibodies (IgM, IgG or both). Serological testing alone depends upon detection of anti‐IgM and increased IgG levels, since specificity of certain assays is not optimal and anti‐HEV IgM itself is not a robust marker for diagnosis. Anti‐HEV IgA antibodies are also used for diagnosis of acute HEV; however, such assays are not widely available. Presence of anti‐HEV IgG helps to determine past HEV infection.^66^

### Antigen assays

Both acute and chronic infections can also be diagnosed by detection of HEV antigen by enzyme immunoassays. Older versions of the antigen assays were not as sensitive as NATs; however, newer assays offer improved and better sensitivity.^67^ Patients with acute hepatitis E may report lower antigen levels than the patients with chronic hepatitis E, with an OD450/630 of >15 (to discriminate between patients with acute or chronic hepatitis E).^68^ Moreover, HEV antigen may exist for several months after ribavirin induced HEV RNA clearance of chronic hepatitis E. The experimental data suggest that the presence of HEV antigen does not necessarily correlate with infectious virions.^68^ In one recent study, it was suggested that glycosylated forms of ORF2 are excreted in the sera of infected patients at high levels; however, infectious virions are associated with the much less abundant nonglycosylated form of ORF2.^69^

### Immunohistochemistry

Immunohistochemistry for HEV ORF2 protein can be used for histopathologic diagnosis of hepatitis E.^70^

## UPDATE OF HEV INFECTION CONTROL

Progress in regulation and prevention have been made to lessen the burden of both chronic and acute HEV in prevalent and nonprevalent countries. Additional studies are still necessary to validate the prominence of preventive measures globally, with prominence on HEV in the public health system.^10^, ^12^, ^13^, ^21^ Prevention actions differ concerning the geographical areas, the dominating genotypes, and their route of transmission. Usually, in low prevalence areas of HEV, the zoonotic transmission dominates, so there is a need to focus on zoonotic risk and food safety. While in areas where the outbreaks are more recurrent, the infection transmits via unhygienic practices and poor sanitation. Therefore, clean drinking water and improving the individual hygiene and sanitation of human wastes can be an effective preventive method.^32^, ^71^ Transmission of HEV through sex, saliva, semen, sweat, and breastmilk is still unclear.^61^ So far, preventive measures for hepatitis E include:

- Virus inactivation is done by destroying the virus in water and food that has to be ingested by either cooking above 90°C of temperature,^10^, ^72^, ^73^ washing the vegetables and fruits using chlorine solutions; doing chlorine disinfection for water supplies and fomites.^74^, ^75^
- Taking care of sanitation, hygiene, and surveillance within the community by treating water supplies and sewage. Moreover, on individual approach washing hands and use of gloves to cook food; for healthcare workers, biosafety guidelines are to be followed, such as basics of wearing specific biosafety clothes during epidemics and dealing with blood sampling‐related procedures during laboratory screening in the blood bank.^13^, ^29^, ^30^, ^31^, ^32^, ^33^, ^34^, ^35^, ^39^, ^40^, ^41^, ^42^, ^43^, ^44^, ^45^, ^46^, ^47^, ^48^, ^49^, ^50^, ^51^, ^62^, ^63^, ^64^, ^69^, ^71^, ^72^, ^73^, ^74^, ^75^, ^76^, ^77^, ^78^, ^79^, ^80^, ^81^, ^82^, ^83^, ^84^, ^85^, ^86^, ^87^
- To accomplish mass vaccination using safe and effective vaccine for vulnerable animals and the public is the need for HEV vaccine control worldwide. WHO endorses further research on the safety, immunogenicity, and efficacy of HEV vaccine.^8^

The necessity for a vaccine is related to the worldwide distribution of HEV, most in the endemic regions and clusters at highest risks such as food workers, patients with chronic liver disease, immunosuppressed patients, tourists to prevalent areas, older adults with more than 60 years of age, pregnant women or those planning pregnancy and children under the age of 2 years. The vaccine is still not recommended globally and as a part of Immunization Practices (ACIP) or emergency immunization.^88^, ^89^ Currently, the HEV239 vaccine is the only vaccine commercially available registered in China since 2011 (Hecolin, Xiamen Innovax Biotech, China), but still not permitted in other countries.^90^ A recombinant vaccine with a three dose (at 0, 1, and 6 months) scheme administered intramuscularly offers protection against HEV4 infection with no evidence regarding protection against other genotypes. It is a recombinant vaccine with 26 amino acids and an extended N terminal with E2, another peptide from the HEV capsid protein. This vaccine method is conceivable as HEV is antigenically well‐preserved, presents only one known serotype that was perceived to be shielding for HEV 1–4 genotypes.^91^, ^92^ The vaccine is supposed to be effective against HEV by counteracting antibodies. A strong T cell‐dependent antibody reaction was noted in mouse models, which was partially ascribed to two T cell epitopes sited in the region of amino acid 533–552 on the HEV capsid peptide after vaccination. This vaccine had showed an efficacy of 97% for preventing episodes of symptomatic acute hepatitis,^90^ with its long‐term efficacy proved at follow‐up.^92^ The vaccine's effectiveness is improved up to 90% after one dose for 1 year and after three doses for 4.5 years. Moreover, the HEV 239 vaccine is harmless for expectant mother and their fetuses, but there is a lack of data for efficacy, specifically in immune‐compromised and chronic liver disease patients.^90^, ^91^, ^92^

## Treatment recommendations

**Figure 3:**
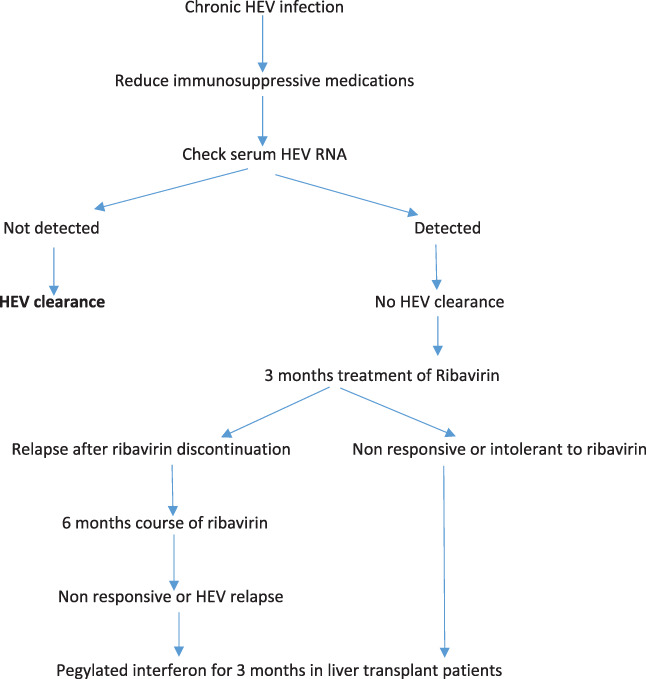
Treatment algorithm for chronic HEV infection (Adapted from European Association for the Study of the Liver.^61^)

There is currently a gap in the practical availability of antiviral drugs or interventions for the patients of chronic hepatitis E, which is essentially characterized by RNA persevering inside the liver of immune‐compromised patients for 3 months. Afterward, it is doubtful that patients realize unprompted viral clearance minus therapeutic mediation.^2^ Currently, three treatment options are available, although no licensed drugs are available, the options available include lessening of immunosuppression as the first choice, use of pegylated IFN‐α (pegIFN‐α) and consumption of ribavirin (RBV) as a monotherapy. (Fig. 3).^30^, ^93^, ^94^, ^95^ RBV monotherapy for 3–6 months or blended with pegIFN‐α is the most frequently used HEV treatment. As its effectiveness in plummeting viral loads has been recognized by many studies involving patients with acute and chronic infections.^30^, ^90^, ^91^, ^92^, ^93^, ^94^, ^95^, ^96^, ^97^, ^98^ Among immunocompromised people, the administration is safe and leads to viral clearance.^96^, ^97^ Moreover, data from in vitro studies advocate that RBV has a reasonable synergistic effect once mixed with pegIFN‐α.^99^ Unfavorably, due to absolute teratogenic physiognomies, RBV is not recommended for pregnant women. Although there are quite a few approaches of action anticipated for RBV and its antiviral outcome against RNA viruses,^99^, ^100^ however, multiple studies have explained that treatment catastrophe can happen. In the case of RBV, these letdowns are related to the choice of viral capability augmenting mutations, executing the intra‐host populace absolute over the influence of lethal mutagenesis projected for RBV. The EASL endorses a tactical lessening of immune system suppression among individuals with obstinate HEV infection as the first intervention strategy.^61^

## Knowledge gaps

As HEV holds physiognomies of a virus using the “hit‐and‐run” approach that supports its genetic evolution. Much exploration is needed regarding the viral particle structure and its receptor(s) that should bring some new information on its tissue tropism and zoonotic spread. Progress in research on contemporary and present challenges, like the epidemiological aspects, the standardization of diagnostic tests, the clinical control of extrahepatic indices, the pathogenesis, prevention in populaces at threat, and the management of chronic hepatitis E may be expedited by analogous advancement to understand the essential traits of biology and the medical inquiry of HEV. The molecular detailing of HEV is insufficient to understand its pathophysiological aspects. There is a lack of much clinical proof about the causal relationship between HEV and its extrahepatic expressions. Understanding model systems is still pending to gain insights for better prevention and control. There is an evident gap concerning the understanding of vaccine safety and efficacy in multi variants of HEV and immunogenicity of HEV 239 along with knowledge about long‐term effectiveness, a period of safety with less than three doses or smaller interims between doses; necessity and scheduling of likely promoter dose. There is also insufficient information related to new lines of treatment and management options. An information gap exists regarding efficiency and cost‐effectiveness of vaccine program in outbreak scenarios.^9^, ^42^, ^47^, ^50^, ^72^, ^84^, ^101^, ^102^

## Research priorities

Future research is necessary to provide evidence regarding the causal link between HEV and extrahepatic diseases, their primary pathogenic mechanisms, assessment of possible management choices, and the identification of the actual burden of disease etc. The few salient research areas that can be prioritized to take the lead over HEV are as follows:

- Sneak into the systematic understanding of critical features of the HEV life cycle from entry to duplication, assemblage, and release
- Analyze the biophysical and biochemical configuration of HEV particles
- Search and study the structural details of the ORF1 polyprotein (pORF1) and protein ORF3 (pORF3) and ORF4
- Identify and define the crucial host factors and tissue tropism
- Explore pathogenesis involved in HEV‐mediated hepatic and extrahepatic manifestations
- Design the direct‐acting or host‐targeting antiviral agents
- Study the efficacy and safety of the HEV239 vaccine
- Generate robust cell culture models supporting infection with all HEV genotypes
- Identify the potential risk areas regarding HEV location among animals and humans and study the changes and evolution
- Generate epidemiological data regarding prevalence, transmission, and overall burden of disease and the study of variations in the curse of infection between or within countries
- Design controllable animal models to study symptoms, innate immune mechanisms, and associated aspects of immunological protection
- Identify the alternative procedure for the treatment of patients that developed an intolerance to current treatment options or being unresponsive at all
- Explore the screening necessities and risks of HEV among blood donors, immune‐compromised and pregnant women

## Conclusion

HEV infection is one of the global health concerns with significant morbidity and mortality. It is one of the leading causes of acute viral hepatitis in developing countries and hepatic failure among pregnant women. HEV usually occurs in industrialized regions, mainly having zoonotic transmission. RBV is a drug of choice in patients suffering from chronic HEV characterized by prolonged viremia that leads to liver cirrhosis and hepatic failure. As per current knowledge and recommendations by WHO & EASL, there are few core essentials to prevent or manage HEV that includes guaranteeing improved sanitation and access to safe food and water based on the country context and epidemic dynamics. However, there are much‐unanswered research and clinical inquiries that need to be explored regarding the evidence for the burden of multiple genotypes of HEV in different geographical distributions, infection sources, and the data for new effective HEV‐specific antiviral drugs and commercial vaccine approval.
